# A Premature Ventricular Contraction Associated With Transient Worsening Pulsus Alternans: A Case Report

**DOI:** 10.7759/cureus.20284

**Published:** 2021-12-08

**Authors:** Angkawipa Trongtorsak, Sittinun Thangjui, Natapat Chaisidhivej, Alisha Sharma, Aekarach Ariyachaipanich

**Affiliations:** 1 Internal Medicine, AMITA Health Saint Francis Hospital, Evanston, USA; 2 Internal Medicine, Bassett Healthcare Network, Cooperstown, USA; 3 Department of Medicine, Einstein Medical Center Philadelphia, Philadelphia, USA; 4 Cardiac Center, King Chulalongkorn Memorial Hospital, Bangkok, THA

**Keywords:** peripartum cardiomyopathy, cardiac alternans, pvc, cardiomyopathy, pulsus alternans

## Abstract

Pulsus alternans is a rare condition characterized by alternation between strong and weak pulses during regular heart rhythm. Although pulsus alternans occurs mostly in severe heart failure, it can also be seen in other conditions that alternate ventricular contraction such as rapid tachycardia and extrasystole. Here, we report the case of a patient with peripartum cardiomyopathy who developed worsening pulsus alternans after a premature ventricular contraction.

## Introduction

Pulsus alternans is a rare finding on physical examination that is characterized by alternation between strong and weak pulses during regular rhythm. This phenomenon can be easily detected during a routine cardiovascular examination by palpating over the arteries. Cardiac abnormalities, severely reduced left ventricular function such as severe cardiomyopathy, and acute myocardial infarction are associated with pulsus alternans [1]. This report presents a case of a young woman with pregnancy-related cardiomyopathy who developed worsening pulsus alternans after a premature ventricular contraction (PVC).

## Case presentation

**Figure 1:**
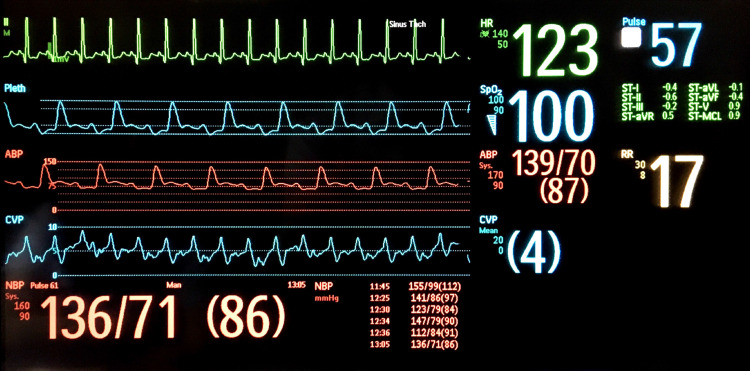
Postpartum day zero. EKG shows sinus tachycardia at 123 beats per minute. The arterial waveform monitor shows alternation of amplitude resembling pulsus alternans, which is also seen in plethysmography. ICU: intensive care unit; EKG: electrocardiogram

**Figure 2:**
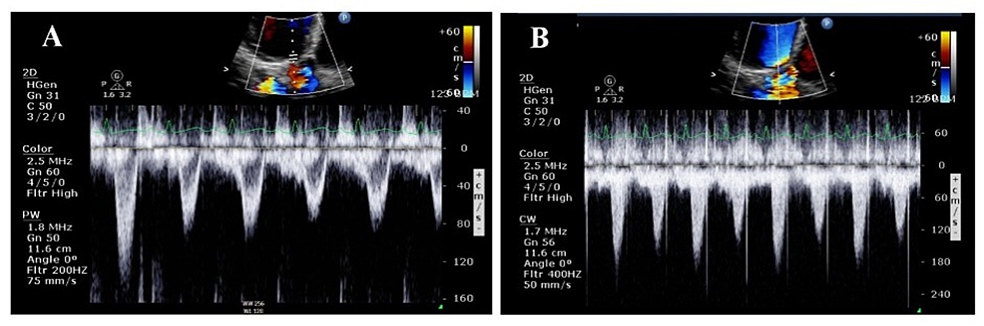
Postoperative echocardiogram. Pulse-wave Doppler of the left ventricular outflow tract (A) and continuous-wave Doppler of transaortic flow velocity (B) show significant beat-to-beat variation of stroke volume and peak velocity, consistent with pulsus alternans.

**Figure 3:**
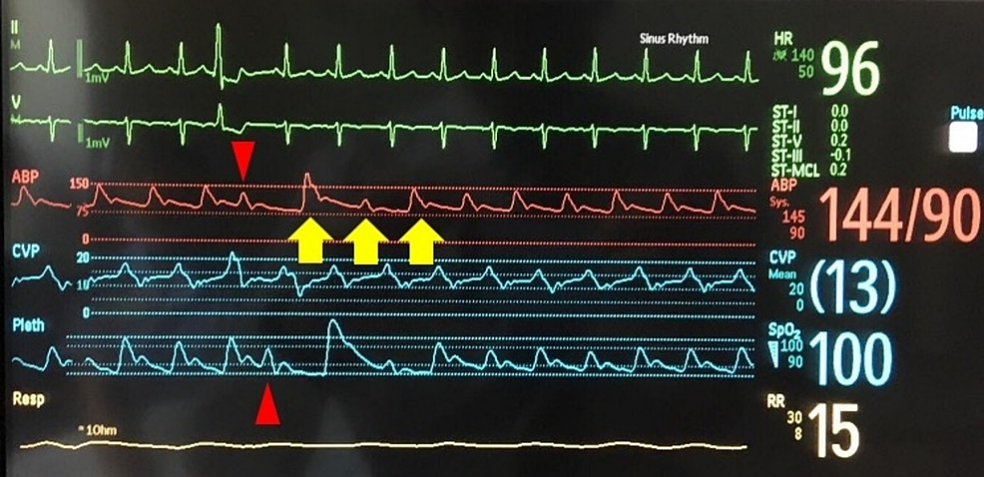
PVC-initiated pulsus alternans in improving heart failure and tachycardia. EKG shows PVCs disrupting the amplitude of the arterial waveform and plethysmographic waveform (red arrow head). This led to transient worsening of the following pulsus alternans (yellow arrow). PVC: premature ventricular contraction; ICU: intensive care unit; EKG: electrocardiogram

**Figure 4:**
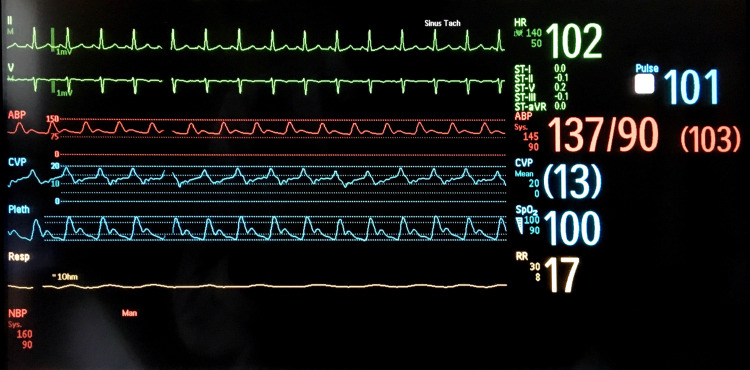
Recovery (postpartum day three). EKG shows sinus tachycardia at 102 beats per minute. Arterial and plethysmographic waveforms show similar amplitude without evidence of pulsus alternans. EKG: electrocardiogram

A 34-year-old woman (gravida 6 para 5) with a history of peripartum cardiomyopathy presented with dyspnea and hypotension one day after an uneventful delivery. The patient was transferred to the intensive care unit (ICU) for acute decompensated heart failure with cardiogenic shock due to peripartum cardiomyopathy. On examination of the radial artery, the pulses were rapid and had abnormal alternating amplitudes, consistent with pulsus alternans. This was also seen in the arterial and plethysmographic waveform, as shown in Figure 1. Electrocardiogram (EKG) showed sinus tachycardia at 116 beats per minute, left ventricular hypertrophy, and occasional ventricular premature complexes (PVCs). Transthoracic echocardiography showed left ventricular ejection fraction (LVEF) of 16% with a significant beat-to-beat variation of stroke volume and peak velocity, consistent with pulsus alternans, as shown in Figure 2. She was treated with dobutamine and diuretics with a resolution of pulsus alternans. Despite the improvement in heart failure and tachycardia, PVCs can initiate transient pulsus alternans (Figure 3). This phenomenon is called post-extrasystolic potentiation (PESP). On the third day of ICU admission (three days postpartum), her acute heart failure improved and PVCs resolved. This resulted in the disappearance of pulsus alternans (Figure 4). She was transferred out of the ICU and discharged home eventually.

## Discussion

Pulsus alternans, first described by Traube in 1872, is a condition characterized by alternation between a strong and a weak heartbeat during regular rhythm [2]. It was originally detected by simply palpating the artery or using a sphygmomanometer. However, the diagnostic sensitivity was low because a difference in the blood pressure between alternating beats can be very small. Plethysmography, arterial catheter waveform, and EKG are more sensitive and precise in diagnosing pulsus alternans.

Pulsus alternans is a part of cardiac alternans, which was categorized by Surawicz and Fisch in 1992 [1]. Although this phenomenon can be found most commonly in severe heart failure, it can occur in other conditions such as myocardial infarction, aortic stenosis, rapid tachycardia, and spinal anesthesia [3-8]. Several mechanisms have been hypothesized to explain this phenomenon. One of the most convincing mechanisms of pulsus alternans was the Frank-Starling mechanism, proposed by Wenckebach in 1910 [9]. He described pulsus alternans as a process in which the strong beat of the alternans leaves a smaller residual end-systolic volume, which, in turn, reduces the end-diastolic volume and the force of the next weak beat. However, this proposed mechanism has limitations as experiments performed to recreate the mechanical alternans on isolated cardiac myocytes showed that the phenomenon also occurred in isolated myocytes despite the elimination of the confounding effects of hemodynamics [10]. This led to the model of the calcium cycling system. The functions of cardiac myocytes include contraction and relaxation. Excitation-contraction coupling and calcium-induced calcium release are the processes of myocyte contraction resulting from the electrical stimulation that leads to high calcium concentration in cytosol and sarcoplasmic reticulum (SR), resulting in myocyte contraction. For relaxation, two mechanisms are involved. Overall, 70% of cytosolic Ca^2+^ undergoes reuptake back into the SR by SR-Ca^2+^ ATPase 2a (SERCA2a) and is expelled out of the cell by the 3Na^+^-1Ca^2+^ exchanger. Alternation of this intracellular calcium handling system was found to induce and abolish the cardiac alternans. The 3R theory describes how alternans arises via an instability caused by the interactions between three critical properties: “Randomness” of Ca^2+^ sparks (baseline microscopic alternans of the subunit of myocyte that is not unified enough to make the whole cell alternans), “recruitment” of Ca^2+^ sparks by neighboring calcium-releasing units (CRUs), and “refractoriness” of CRUs. Faster heart rate increases the refractoriness of myocytes by delaying Ca^2+^ release and Ca^2+^ reuptake, resulting in overall calcium accumulation. This causes CRUs to synchronously fire on every other beat, which leads to whole-cell macroscopic alternans and cardiac or pulsus alternans. These findings suggest that pulsus alternans can be provoked by increasing heart rates that exceed a certain threshold [8]. The threshold can be very high in a normal heart but is significantly lowered in conditions such as heart failure, hypothermia, hypocalcemia, hypercapnic acidosis, and ischemia. Extrasystole or PVCs and a pause can also induce transient pulsus alternans that spontaneously resolve within a few beats in the absence of rapid heart rate or other induction methods [11]. PVC-induced pulsus alternans was thought to arise from abnormal calcium homeostasis which disrupts the calcium cycling system and causes cardiac alternans [12]. PVCs also produce a PESP, a phenomenon that increases the contractility of the beat following an extrasystole which leads to the initiation of pulsus alternans [11].

In the clinical context, detecting pulsus alternans is a useful predictor of poor outcomes in acute heart failure patients. Pulsus alternans is associated with a twofold increase in mortality at 10-month post-discharge from an acute ICU hospitalization due to heart failure, regardless of ejection fraction [13]. Moreover, patients with idiopathic dilated cardiomyopathy, in which pulsus alternans can be induced by fast heart rate, have poorer outcomes compared to those without pulsus alternans [14].

## Conclusions

Pulsus alternans is associated with poorer outcomes in heart failure patients. However, it is unclear whether pulsus alternans will play a significant role in managing patients in the future. Nevertheless, the observation of pulsus alternans has led to investigations of the pathophysiology of the heart and has opened doors to more important clinically significant findings.
